# Telomeres and telomerase in Sarcoma disease and therapy

**DOI:** 10.7150/ijms.97485

**Published:** 2024-08-06

**Authors:** Jin Huang, Yan Feng, YangJing Shi, Weilin Shao, Genshan Li, Gangxian Chen, Ying Li, Zuozhang Yang, Zhihong Yao

**Affiliations:** 1 Department of Cancer Research Institute, The Third Affiliated Hospital of Kunming Medical University (Yunnan Cancer Hospital, Yunnan Cancer Center), Kunming, Yunnan, 650118, China.; 2 Bone and Soft Tissue Tumours Research Centre of Yunnan Province, Department of Orthopaedics, The Third Affiliated Hospital of Kunming Medical University (Yunnan Cancer Hospital, Yunnan Cancer Center), Kunming, Yunnan, 650118, China.; 3 Kunming Medical University, Kunming, Yunnan, 650500, China.

**Keywords:** sarcoma, osteosarcoma, telomeres, telomerase, telomere shortening, TERT promoter mutation

## Abstract

Sarcoma is a rare tumor derived from the mesenchymal tissue and mainly found in children and adolescents. The outcome for patients with sarcoma is relatively poor compared with that for many other solid malignant tumors. Sarcomas have a highly heterogeneous pathogenesis, histopathology and biological behavior. Dysregulated signaling pathways and various gene mutations are frequently observed in sarcomas. The telomere maintenance mechanism (TMM) has recently been considered as a prognostic factor for patients with sarcomas, and alternative lengthening of telomeres (ALT) positivity has been correlated with poor outcomes in patients with several types of sarcomas. Therefore, telomeres and telomerases may be useful targets for treating sarcomas. This review aims to provide an overview of telomere and telomerase biology in sarcomas.

## 1. INTRODUCTION

Cancers derived from mesenchymal cells are called sarcomas^1^. Sarcomas are a cluster of different nasty excrescences that occur in soft tissues and bone^2^. Histologically, osteosarcoma (OS) and soft tissue sarcoma (STS) are considerably different^1^, and they comprise 1% of all cancers. The incidence of sarcomas is 1-5 cases per 1 million people^3^. While making up just 1% of all cancers in humans, sarcomas are the second most common solid tumor type in children and adolescents^4^. A total of 20% of juvenile and 1% of adult solid malignancies are bone and STS malignancies, respectively. There are more than 50 different sarcoma subtypes, 50% of which affect the musculoskeletal system and arise in the extremities. Each subtype has great morphological and phenotypic heterogeneity and is highly diverse^5^. The skin, organs, and other soft tissues such as adipose tissue, muscle, nerve sheath, and blood vessels are frequent sites of STS development^6^. OS, Ewing sarcoma (EWS), and chondrosarcoma (CS) are the most common bone sarcomas (BS), accompanied by metastatic illness^7^. OS arises from osteoblastic cells. ES arises from round cells of the bone marrow, primitive neuroectodermal cells, or neural crest cells. CS are the most common types of bone cancer, which most likely originates from transformed chondrocyte cells developing from mesenchymal stem cells (MSC)^8^. OS usually develops in areas where bones grow quickly, particularly at the ends of long bones^9^.

It is difficult to make diagnosis for the variety types of sarcomas. Sarcomas occur in various forms, and each subtype typically exhibits distinctive manifestations. And, the same form of sarcoma may occur in different organs with greatly different clinical behaviors^6^. Different subtypes of sarcoma have different diagnostic criteria. While, the prognosis for most patients with sarcoma is poor. The 5-year survival rates of patients with non-metastatic and metastatic OS are 60% and 30%, respectively, owing to strong invasive capacity and primary metastasis^10^. Patients with EWS or CS have similar outcomes. To improve the survival rate and prognosis of patients and achieve better treatment results, it is essential to explore the pathogenesis of different sarcomas and screen targeted drugs. Repeated nucleoprotein structures known as telomeres are present at the boundaries of chromosomes. They are crucial for chromosomal end preservation, inhibition of DNA damage response (DDR), and preservation of genomic stability^11^. Tumor development may result from an anomaly in the telomere structure. One of the factors that causes cancer is telomere length. Both long and short telomeres maybe stimulate the growth of cancer. Telomere maintenance is an essential phase in carcinogenesis, including telomerase activation and selective telomere extension. It is interesting that telomere maintenance is frequently positive in many subtypes of sarcomas frequently linked with poor prognosis. The primary mechanism is telomerase overexpression. However, a small percentage of malignancies use a homology-directed repair-mediated telomerase-independent ALT mechanism^12^. The usage of ALT is common in some cancer types, such as OS and glioblastoma (GBM), occurring in approximately 5-15% of all human malignancies^13^. Although, RNA component called telomerase RNA (TERC) is widely expressed in healthy mortal physical cells, telomerase reverse transcriptase (TERT) is epigenetically suppressed, and its expression is thought to be a limiting factor in telomerase activity. Reactivated telomerase is estimated to be present in over 90% of human malignancies^14^. Telomerase activity is often elevated in bone and STS. Aberrant expression of TERT, a component of telomerase, is strongly associated with sarcomas^15^. Therefore, the telomeres and telomerases have important potential in treatment of sarcomas.

## 2. TELOMERES BIOLOGY IN SARCOMA

### 2.1. STRUCTURE OF TELOMERE

The last component of the linear chromosomes is the called telomeres^16^. The six-protein shelter in complex and telomeric DNA form complexes to form human telomeres^17^. Mammalian telomeres are made up of tandem TTAGGG DNA repeats that span many kilobases, about 10∼15 kb in humans and 25∼50 kb in mice^18^, ^19^. The telomere has a unique DNA design that consists of two components in extension to its repeating DNA property. A lengthy double-stranded DNA (dsDNA) region that ends in an unattached 3′ tail, was understood as the G-overhang^20^. The recurring sequence of nucleotides TTAGGG makes up the 3′ ends of chromosomes^21^. The coplanar arrangement of four guanine bases known as a "G-quartet" is held together by hoogsteen hydrogen bonds and kept stable by physiologically plentiful Na^+^ or K^+^ cations. G-quadruplexes can be created by stacking G-quartets depending on the number of strands involved, strand direction, and changes in loop size and sequence. These structures can adopt a broad range of topologies^22^. A 30∼400 base 3' overhang of the G-rich strand gives telomeres their distinctive appearance. What is known as a telomeric (T) looping procedure and a displacement (D) loop are created when the G-strand overhangs, fold back, and invades the double-stranded telomeric area. It has been suggested that the T-loop topology can shield chromosomal ends from telomerase activity, DNA repair, and degradation^18^, ^19^. DNA TTAGGG sequences were linked using shelterin^23^. The six subunits that make up the shelterin complex attach to telomeric DNA directly in three of them (TRF1, telomere repetition bound factor 1; TRF2, telomere repetition bound factor 2; POT1, the protective of telomere protein 1), and three of which mediate the interaction between components (TIN2, TRF1, and TRF2 interaction Nuclear Protein 2; RAP1, Repressor-Activator Protein 1; TPP1, and TIN2 Organized Protein)^24^. TRF1 and TRF2 have different surface interactions when bound to TIN2. Additionally, TIN2 binds to TPP1, which then binds to POT1 (POT1a and POT1 in mice). The six-subunit complex is formed when Rap1 binds to TRF2. Despite many documented alterations in the subunits, the binding surfaces between shelterin subunits have a variety of structural characteristics and most likely do not include post-translational modifications^25^. POT1 engages with the single-stranded telomeric overhang, whereas TRF1/2 interacts with double-stranded telomeric DNA. Through protein-protein interaction, the final three proteins connect to the telomeres^26^.

### 2.2. FUNCTION OF TELOMERES

To shield chromosomal tails from the DNA repair machinery, telomeres, which are dynamic nucleoprotein structures, create protective caps^27^. These sequences come together to create a cap that serves at least two protective purposes: (1) it enables cells to discriminate between double-strand breaks and chromosomal ends and (2) it prevents chromosomes from being destroyed, recombined, and fused end to end^28^. Loss of the cap renders telomeres indistinguishable from double-stranded breaks, resulting in checkpoint capture, telomere fusion, and unregulated deterioration^29^. The likelihood of genomic instability and tumorigenesis are considerably increased by this process^30^. Short telomere cells restart and accelerate transformation phenotypes if DNA damage response barriers, such as p53 loss, are overcome. In this situation, telomerase activity enables complete malignancy possible^31^. DNA polymerases' traits include the restriction of DNA replication to the sequence from 5' to 3' and the requirement of a primer, frequently RNA, to initiate the synthesis of DNA. When the terminal RNA primer is withdrawn, a gap at the 5' tails of freshly duplicated strands is left that cannot be addressed by a conventional DNA polymerase. As a result, when DNA replication cycles continue, telomere length gradually decreases, ultimately leading to the loss of genetic information^29^.

#### 2.2.1. FUNCTION OF TELOMERE LENGTH

The telomeric environment, genetic variables, and related telomeric proteins such as telomerase affect the length of telomere^32^. According to a cross-sectional study, women who reported feeling stressed had telomeres approximately a decade shorter than those who reported feeling less stressed. Environmental variables (such as smoking, alcohol, drugs, and medicines) and lifestyle factors (such as obesity, exercise, and psychological factors) can be modified to enhance health outcomes, which may be partially attributed to telomere maintenance^33^. Since telomere length gradually decreases with each dividing cell in its lack of extension, telomere length is highly variable, regardless of whether it's within an individual cell. And it functions as a "molecular clock" of the proliferating lifetime of primordial cells^20^. A DNA damage reaction that causes senescence, persistent cell cycle arrest, or apoptosis in cells is signaled when only one or a tiny number of telomeres pass through an important barrier, potentially approximately 100 pairs of bases^34^. Inactivation of the tumor-suppressor proteins, p53 and Rb, can stop this process. Mutations in TP53 and RB1 were positively associated with telomere length^35^. Cells with severely short telomeres can continue to divide longer when p53 and RB are inactive. However, this leads to telomere crisis, a time when cells begin to die and the genome becomes unstable^36^. Numerous clinical problems, including a higher chance of developing schizophrenia, depression, cancer, diabetes, heart failure, cardiovascular illness, T-cell immunodeficiency, cognitive decline, and early death, are associated with a short telomere phenotype. However, melanoma, adult glioma, and chronic lymphocytic leukemia seem to be at an increased risk due to longer telomeres. In similar studies of pediatric malignancies, longer telomeres have been linked to an increased likelihood of OS and neuroblastoma^28^. In vertebrate models, there is strong evidence that long telomeres are predisposed to cancer. Long-telomere mice consistently performed poorly when the ability of oncogenes to cause tumors was examined in mice with short and long telomeres, such as when Myc or KRAS was overexpressed. These mice developed more aggressive tumors and had lower survival rates^37^. Telomere length is a significant limiting factor in how well telomeres can perform. To retain the ability of a cell to replicate for an extended period, telomere length must be maintained. There are two basic ways to accomplish this, either by the activity of the specialized enzyme telomerase or by ALT, which is mediated by homologous recombination^38^. Cancer cells can reproduce indefinitely because of telomere extension^39^. To prevent telomere shortening, protect telomeres from DNA damage repair mechanisms, and prevent senescence and/or apoptosis caused by telomeres, cells must develop TMM during carcinogenesis. Thus, telomere upregulation is crucial for cancer development^40^. Human carcinomas contain telomeres that are noticeably shorter than those in normal tissues, and early stages breast tumors have been found to have telomere-to-telomere fusions. Patients with early stage chronic lymphocytic leukemia are more likely to have telomere dysfunction than those in the later phases of the disease, which are marked by telomere-to-telomere fusion^41^.

#### 2.2.2. FUNCTION OF SHELTERIN

The capacity to sustain the shelterin complex is now widely acknowledged to be lost when telomere length is dangerously low. The release of the shelterin complex's inhibitory effect on the reaction to DNA damage pathways causes the cell phase to leave G1 and enter G0^38^. Telomere stability is guaranteed by crucial and unique tasks performed by the shelterin complex. TRF2 is necessary, for instance, to generate T-loops and maintain DDR and non-homologous end-combining inhibition through ataxia telangiectasia mutated (ATM). Whereas POT1 and TPP1 work together to attach to the single-stranded 3′ overhang and prevent the recruitment of replication protein A to decrease ATR-mediated DDR, TRF1 has a significant impact on the regulation of telomeric DNA replication. Because TIN2 stabilizes the interactions of TRF1 and TRF2 with telomeric DNA and connects the TPP1/POT1 heterodimer to TRF1 and TRF2, it is crucial for shelter complex stability. RAP1 interacted with TRF2 to enhance telomeric DNA-specific binding^42^. According to recent studies, mutations in the shelterin complex may cause a variety of malfunctions, eventually contributing to the development of human tumors. It is noteworthy that shelterin gene mutations in melanoma, glioma, sarcoma^41^, cardiac angiosarcoma (CAS), Li-Fraumeni-like syndrome (LFLS), colorectal cancer, Hodgkin's lymphoma^37^, mantle cell lymphoma, and parathyroid adenoma reveal extensive possible importance in the development of non-epithelial cancer^43^. Thirteen carriers of C4 or C5 variants were found in four of the six canonical genes in the shelterin complex: POT1 (6), TERF1 (2), TINF2 (3), and TERF2IP (2). Furthermore, the cancer incidence was similar to that of TP53, but higher in the families of shelterin probands having C4 or C5 variants. In probands with sarcoma, the relative leukocyte telomere length (RLTL) gradually decreased over time, independent of exposure to radiation or chemotherapy. The RLTL of shelterin variant carriers was longer than that of other cohort members, despite their similar ages at first cancer diagnosis. Sarcoma is more likely to occur than other epithelial malignancies because of heritable abnormalities in telomeres and mitotic functions. Most sarcomas were genomically unstable. Replication stress caused by ALT results in genomic instability, which sends a signal to TP53, a gene closely associated with sarcoma susceptibility. 0.8% sarcoma probands had TP53 mutations, whereas 3.2% had C3 to C5 polymorphisms in the shelterin complex 15. It was shown that a missense mutation (p.R117C) in the POT1 gene was the cause of CAS^44^. Angiosarcoma (AS) exhibited a POT1 mutation 11 times more frequently than other tumors. Patients with LFLS and spontaneous AS have been shown to carry five harmful POT1 variations. Each conserved domain contained POT1 mutations. Given the high frequency of POT1 mutations in AS, it is likely that a key factor contributing to the formation of AS is the loss of POT1 function. Therefore, POT1 analyses should be prioritized in AS sequencing investigations^45^. POT1 mutant proteins lose their ability to bind single-stranded DNA and TPP1. When telomeres are abnormally long, function is lost^46^. ATR-dependent DNA damage signaling is activated by both damage response malfunction and POT1 gene function inhibition 47. A study discovered that, in comparison to POT1 wild-type, POT1 PVs had a considerably increased incidence of sarcoma and melanoma 48. The broad range of POT1 gene variations that cause cancer in many tissues indicates the universal significance of these genes. POT1 analysis should be regarded as a standard diagnostic procedure for these malignancies^46^.

### 2.3. TELOMERES AND SARCOMAS

Liposarcoma (LPS), leiomyosarcoma (LMS), and undifferentiated pleomorphic sarcoma (UPS) are the three most prevalent types of STS 49. Sarcomas and gliomas have longer telomeres than other cancers, whereas tumors have shorter telomeres than normal tissues^35^. The risk of STS is greatly increased by longer telomeres in peripheral blood leukocytes^50^. The chances of lymphoma, OS, and STS have been found to be higher in patients with a long leukocyte telomere length. It has been shown that the largest correlation was shown in gastrointestinal stromal tumor (GIST), where a 2.2-fold higher risk was associated with longer LTL. In LMS, it was significant, but not for LPS or angiosarcoma. Unknown biological factors underlie the association between increased cancer risk and longer LTL. Recent studies have shown that both short and long telomeres promote cancer development. Telomere disruption may contribute to the development of cancer. Telomeres that are abnormally short can promote chromosomal end-to-end joining, which can result in genomic instability and cancerous changes. In contrast, extremely long telomeres may increase the risk of cancer development by promoting prolonged cell division and delaying cellular senescence and apoptosis, creating a setting that favors the development of genetic defects. The unique tissue genesis of sarcomas may be another biological factor contributing to the substantial correlation between extended LTL and high STS risk. Sarcomas are malignant tumors originating from the mesenchyme, in contrast to carcinomas, which start in epithelial cells. While mouse and pediatric human malignancies are mostly sarcomas and lymphomas, adult human tumors are primarily epithelial carcinomas^51^. To enable immortalization, telomere shortening forces tumor cells to trigger a TMM^52^, ^53^. Sarcomas that often test positive for ALT include OS, LMS, LPS, and undifferentiated pleomorphic sarcomas (malignant fibrous histiocytoma)^54^. Inherited abnormalities in telomeres and mitotic function raise the likelihood of sarcomas in comparison to most epithelial malignancies. Chromosome integrity depends on mitosis and telomere maintenance. Sarcomas are primarily characterized by genomic instability and use an alternative mechanism called ALT. Through replication stress, ALT induces genomic instability and TP53 signals, which are closely associated with sarcoma susceptibility. TERF1, TINF2, and POT1 are associated with sarcoma^15^. By continuously transplanting Pot1b-/- in mice, POT1b's function in controlling the maintenance of telomere length was examined. Early Pot1b-/- tumors initially had shortened telomeres, whereas late-stage Pot1b-/- cells exhibited significantly elongated telomeres, which are recognized as DNA damaged by the replication protein A (RPA) complex. Telomere extension is a result of telomerase recruitment being encouraged by the RPA-ATR-dependent DNA damage response at telomeres. POT1b inhibits the DNA damage response by unfolding G-quadruplex structures within stretched telomeres, which POT1a is not able to do. This suggests that the phenotypes observed in human cancers with POT1 mutations may be caused by similar mechanisms^55^. ALT is a more important TMM in LMS than telomerase activation. 31 LMS, 53% tested positive for ALT expression. Additional research has demonstrated a correlation between ALT-positive LMS and tumor necrosis, poor differentiation, high FNCLCC grade, and epithelioid/pleomorphic cell shape^54^. Most ALT cell lines exhibit signs of p53 pathway malfunction, most frequently due to TP53 mutations or viral oncoprotein-mediated p53 degradation. Mutations that inactivate ATRX are frequently observed in ALT malignancies; however, in zebrafish, ALT tumors are produced by both ATRX and TP53 knockouts, but ATRX knockouts do not activate ALT. Finally, telomere replication stress and dysfunction cause the ALT phenotype; however, when p53 is functional, it causes growth arrest and/or apoptosis. Although p53 pathway changes are frequently observed in ALT cells, p53 inactivation by itself is unlikely to cause ALT alone because most TP53-mutant malignancies are telomerase-positive (TA+)^56^. ALT-specific molecules, such as C-circles, are the only known molecules. For rolling-circle amplification, the C-circles are self-primed telomeric C-strand templates. Diverse centrifugation methods have been used to separate extracellular vesicles from the growth media of lung adenocarcinoma, GBM, neuroblastoma, OS, and STS cell lines. Of these, it was discovered that the exosome fractions from 11 ALT+ cancer cell lines included C-circles, whereas the normal fibroblast strain and the eight telomerase-positive cancer cell lines did not have any extracellular vesicles in their extracellular fractions. C-circles secreted by ALT+ cancer cells in exosomes exist as durable blood-based biomarkers and are possible clinical diagnostics for ALT activity^57^. GID4 and RAD51B are involved in telomere elongation in sarcomas^58^. The major outcome examined was the risk of mortality, which was computed as the number of deaths divided by the total number of participants during follow-up in ALT+ vs. ALT- individuals. These findings suggest that ALT is associated with a higher risk of mortality in patients with sarcomas. ALT levels should be considered when designing prospective therapies and performing precise prognostic stratification of these neoplasms^59^.

#### 2.3.1. TELOMERES AND OS

Children, teenagers, and young adults are most frequently affected by OS^60^. 10%-15% of patients with newly diagnosed OS detected the metastatic disease, mostly in the lungs^61^. ALT is a common finding in 5-15% of human malignancies and is frequently linked to a poor prognosis, especially in OS and GBM^62^. In particular, the highest incidence of any tumor that has been studied exhibits ALT features in over 60% of OS cases^52^. Candidate gene analysis and genome-wide association studies (GWAS) have revealed potential risk variations in European-heritage individuals. In a multi-ethnic sample of children who were non-Hispanic white, Hispanic, African-American, or Asian/Pacific Islanders, an independent multi-ethnic dataset investigated the previously documented association between genetic susceptibility to longer LTL and the risk of OS. The result revealed that the highest impact was observed in Hispanic participants, and that genetic predisposition to prolonged LTL is a risk factor for OS^63^.

#### 2.3.2. TELOMERES AND STS

Many different subtypes of LPS exist, each with its own characteristics^64^. In dedifferentiated liposarcomas (DDLPS), alternative telomere lengthening is a predictive indicator of poor prognosis. Telomerase activation is the major method of telomere maintenance in myxoid liposarcomas (MLS), whereas ATL is used for pleomorphic and DDLPS^65^. Telomeres are promising therapeutic targets for DDLPS. Targeting G4 structures may be an attractive and novel treatment for DDLPS and merits further research. Thus, G4 ligands are promising therapeutic agents for this condition^66^. LMS, accounts for 10% of all STS and affects individuals of all ages. It is a malignant tumor of smooth muscle origin that most frequently affects the uterus, retroperitoneum, and major blood vessels. Approximately 40% of the patients eventually develop local recurrence and/or metastasis. Patients with disseminated LMS are typically uncurable condition^67^. LMS that tested positive for ALT had tumor necrosis, epithelioid or pleomorphic cell shape, poor differentiation, and a high FNCLCC grade^54^.

## 3. TELOMERASE BIOLOGY IN SARCOMA

### 3.1 STRUCTURE OF TELOMERASE

In order to emphasize its capability to add telomeric sequence repeats, telomerase was given the name "telomere terminal transferase" when it was first identified in 1985^68^. Telomerase is a ribonucleoprotein (RNP) complex that includes TERT, TERC, and several auxiliary proteins^69^. By catalyzing the extension of the 3′ end of the telomeric DNA, telomerase keeps the length of the telomere constant. During S phase, both components functionally associate in the nucleus with the temporary assistance of a number of other elements^70^. These two molecules mediate the canonical function of telomerase. After TERC attaches to the single-stranded overhang at the telomere, a hexameric repeat is added and used as a template by TERT for reverse transcription. Telomere elongation is made possible by reverse transcription and the repeated translocation of TERT and TERC to the 3′ end of the chromosome^71^.

#### 3.1.1. HUMAN TELOMERASE REVERSE TRANSCRIPTASE (hTERT)

In humans, TERT is located on chromosome 5p15.33. The TERT gene has a 260 bp promoter core and is 42 kb long with 15 introns and 16 exons. Five to nine exons encode the reverse transcriptase domain. 22 different isoforms of the TERT transcript isoforms have been created^72^. TERT is composed of an RNA-binding domain (RBD), conserved reverse transcriptase (RT), a carboxy-terminal element (CTE), and an additional reverse transcriptase. Together, these domains constituted the TERT ring structure. In addition, it has a lengthy linker that connects the RBD to the telomerase amino-terminal domain (TEN)^73^, ^74^. Surprisingly, the TERT regions of promoters exclude the TATA and CAAT boxes while being rich in binding structures for numerous transcription variables, including the MYC oncogene (E-box) and Sp1 (GC box)^75^. Secondary DNA structures, called G-quadruplexes, are formed when guanine nucleotides are repeated. Within its core promoter, the hTERT promoter contains nine putative tandem G-quadruplex-forming regions^76^. Telomerase activity was constrained using TERT^77^. TERT is a specialized reverse transcriptase that differs from conventional reverse transcriptases in promoting template realignment to continue the synthesis of numerous DNA repeats without separating them from the telomere. TERT is a widely recognized therapeutic target because it is expressed only in cells with telomerase activity and not in a large number of healthy somatic cells^78^. Increasing TERT copying quantity, TERT gene amplification, genomic rearrangements, long-range interactions, and numerous point mutations in the TERT gene's promoter region include known methods of TERT reactivation that can boost TERT mRNA expression and TERT protein synthesis^79^. It is currently known that 19 percent of malignancies include single-base mutations that produce de novo binding sites in the proximal hTERT promoter for the ETS group of transcriptional components, resulting in gene reactivation. The T-INT1 region of the genome, which is 260 kb upstream and contains multiple binding motifs for GA-binding proteins (GABP) and members of the ETS transcription factor family, is necessary for the long-range chromosome connection necessary for the reactivation of the modified hTERT agents for promotion. By activating the molecular mechanism of WT-hTERT, researchers discovered that WT-hTERT transcription is active in CRCs and requires the transcription factor JunD, which belongs to the AP-1 family. The creation of Sp1-Sp1 tetramers within the proximate WT-hTERT promoter and a distant regulatory chromatin region, which we refer to as tert-interacting region 2, is made possible by increased amounts of JunD that foster a favorable chromosomal structure^80^. In contrary to wild-type promoters, TERT transcription is driven by the enrichment of active histone marks, which are mediated by long-range chromatin interactions, and the transcription element GABPA is exclusively recruited to mutant TERT promoters^81^. TERT was found to be expressed in 73% of 6,835 tumors and was linked to TERT point mutations, rearrangements, DNA amplifications, and transcript fusions, in addition to being predictive of telomerase activity. TERT promoter methylation is another route of deregulation of TERT expression^35^. Genomic analysis revealed, for the first time, the presence of TERT amplification and ATRX mutations in a subset of high-grade and dedifferentiated CS (20%), along with TERT promoter mutations. These abnormalities in telomere genes co-occurring with IDH1/IDH2 mutations and CDKN2A/2B loss or TP53 mutations suggest the potential associations and synergistic effects of these genes on CS progression^82^. Regarding TERT mutations, tumors with IDH1 or IDH2 mutations exhibit notably distinct genetic pathways and consequences. Thus, prognosis and clinical surveillance may be guided by diagnostic testing for TERT mutations^83^. Myxoid/round-cell LPS had a much higher enrichment of mutations in the TERT promoter (88%). TERT promoter mutations were not linked to tumor grade^84^. TP53/pTERT abnormalities may be used as molecular markers to indicate the origin of cells from hepatocellular carcinoma or mixed hepatocellular cholangiocarcinoma, as genetic alterations are maintained during sarcomatous transformation^85^. To investigate the previously unexplored genetics of these tumors, targeted next-generation sequencing and DNA copy number analysis through comparative genomic hybridization were performed on 40 pleomorphic dermal sarcomas. More than half of the samples had verified TERT promoter mutations^86^. Sanger sequencing was used to examine 116 patient samples identified as having 22 different histological subtypes of bone and STS, to check for TERT promoter mutations. Mutations in the TERT promoter were found in seven distinct sarcoma subtypes, with an overall incidence of 9.5%^87^. The study showed the TERT promoter hotspot mutation was not significantly correlated with clinicopathological characteristics or overall or metastasis-free survival. TERT aberrations may arise early in life and contribute significantly to tumor development, although they are not predictive factor^14^. An identical mutation was observed in both components of the five matched conventional CS and in high-grade components with TERT mutations^88^. A multi-center database study was conducted to describe the genomic landscape of gliosarcoma (GS) which found that 92% of cases of GS have mutate in the TERT promoter 89.

#### 3.1.2. HUMAN TELOMERIC RNA COMPOENT (hTERC)

The TERC gene on chromosome 3q26 encodes a part of the human telomerase RNA component (hTERC or hTR)^90^. The length of TERC, which ranges from 150nt in ciliates to >2000nt in certain yeasts, is extremely diverse in contrast to the comparatively conserved TERT. The rapid development of the noncoding RNA (ncRNA) TER, explains this. The template/pseudoknot domain (t/PK), which generates a circular shape with the template and pseudoknot, and the stem-terminus element (STE), which comprises a hairpin, are the two sections of TERC preserved for interaction with TERT^73^. All hTERCs contain a brief sequence complementary to the telomeric TTAGGG hexanucleotide repeat sequence, although they differ in size and sequence depending on the species. However, the structures of hTERCs are highly conserved. The hTERC is a short (451nt as opposed to >1000nt in yeast)^30^. The G-rich region at the 5′ end of TERC, the RNA component of telomerase that houses the template sequence for completely new production of telomeric repetitions, has the potential to fold into a G-quadruplex. The creation of a helical region vital for determination of the template border in mammalian telomerase is thought to be hindered by this structure, which is also thought to protect TERC from degeneration during the initial phases of telomerase ribonucleoprotein synthesis^91^. In contrast to TERT, the expression of which is strictly controlled, TERC is widely expressed in both healthy and malignant tissues. The 5′ pseudoknot structure that interacts with TERT contains a region that functions as a template for telomere synthesis, making up the primary functional domain of TERC^78^. The primary function of TERC is to aid the extension of telomere by acting as a scaffold and template for telomerase RNP^92^. The 3' H/ACA short nucleolar RNA-like domain of TERC is composed of the ACA box at the 3' terminal tail and the H box in the single-stranded hinge region after the 5' hairpin. DKC1, GAR1, NHP2, and NOP10 are examples of H/ACA accessory proteins in the telomerase complex that bind to the TERC H/ACA motif to stabilize it and improve the TERC-TERT interaction. Pseudouridylation of TERC is the mechanism by which these H/ACA proteins stabilize TERC 93. Although TERC is found in germline tissues and telomerase-positive tumor cells, it is also expressed in normal somatic cells. Furthermore, it is expressed in tumor cells that lack telomerase and uses the ALT route to preserve telomere length. Research on tumor grade, aggressiveness, progression, and differentiation of lesions into high- and low-risk groups has demonstrated the clinical significance of TERC amplification in cancer^92^.

### 3.2 FUNCTION OF TELOMERASE

In most eukaryotes, telomerase is a crucial reverse transcriptase required for the maintenance of linear chromosomes^94^. Each time a cell divides, telomeres shorten; however telomerase prevents this from happening^95^. Cell immortality, or the ability of cells to multiply perpetually, is a property conferred by telomerase^96^. Telomeric DNA complies with the TERC once telomerase and telomeres join. Then, telomerase moves its RNA template to the new 3' telomeric end without separating from the telomere and adds one telomere repeat de novo to the 3' DNA end^41^. Cell proliferation is facilitated by the well-described canonical function of telomerases, which is to preserve the integrity of chromosomal end structures (telomeres)^78^. A thorough understanding of telomerase assembly and control has direct biological implications, because telomerase is frequently elevated in human malignancies. In addition, telomerase is related to a group of RNP enzymes that regulate a wide array of cellular functions, including the synthesis of proteins on the ribosome and processing of messenger RNA by the spliceosome. Telomerase also functions as an effective model system for comprehending the fundamental principles that regulate RNP structure and function, because each of these crucial RNP complexes depends on the precise interdependence of protein and RNA components^97^. While somatic cells lack telomerase activity, stem cells and most cancer cells do^98^. Cancer is characterized by cell proliferation, making treatment measures desirable. Additionally, because the enzymatic activity of telomerase ensures that both primary and metastatic tumor cells have an endless capacity for cell division, it appears to be a natural target for the development of cancer therapies^99^.

#### 3.2.1. FUNCTION OF TERT

The TERT gene is tightly suppressed at the transcriptional level in a large number of differentiating human cells, which is caused by telomerase silencing in these cells despite the widespread expression of TERC in human cells. A key mechanism for telomerase activation throughout the tumorigenic phase is the de-repression of TERT, which induces TERT expression^100^. Chromosome ends are added to a six-base DNA repeat via hTERT reverse transcriptase activity, which prevents them from shrinking because of subsequent cell division^101^. In human somatic cells, telomerase activity is downregulated when TERT is silenced^102^. Critical telomere attrition eventually causes DDR, which induces cell cycle arrest and senescence of replication or apoptosis through p53 or RB tumor suppression mechanisms^103^. Most malignancies are characterized by elevated TERT^27^. TERT's abnormal TERT expression is linked to 85-90% of the malignancies that have been studied, despite the fact that it is ordinarily undetectable in somatic cells (apart from stem cells). For over 20 years, TERT has been recognized that TERT is a target for cancer treatment because of its exclusive expression in cancer cells^104^. Through a variety of processes, including amplification of the hTR-encoding genes TERC and TERT, telomerase activity increases in cancer. The development of non-coding mutations in the TERT promoter is the best-understood mechanism by which malignancies boost TERT transcription^36^. Numerous human cancer types, such as thyroid carcinomas, thyroid squamous cell carcinomas, gliomas, renal cell carcinomas, melanomas, urothelial carcinomas, oral squamous cell carcinomas^105^, bladder cancer, melanoma on the skin, basal cell carcinoma, oligodendroglioma, hepatocellular carcinoma^36^, and LPS^106^ exhibit hTERT overexpression. The cellular location, transcriptional control, and holoenzyme assembly are believed to contribute to the regulation of hTERT activity^76^. Heterozygous point mutations in the TERT promoter trigger TERT transcription. Beyond the TERT translation start location, at positions -124 and -146, correspondingly, the two most frequent activating mutations are both cytosine to thymine mutations (-124C>T and -146C>T)^107^. In mitosis, CDK1 phosphorylates hTERT, and this phosphorylation event impacts RNA-dependent RNA polymerase activity rather than hTERT function at telomeres^108^. Interestingly that in tumors with a high incidence, such as lung, breast, prostate, and colorectal cancers, TERT promoter mutations are either absent or were found only extremely rare. Overall, compared to tissues with strong self-renewal (such as bone marrow and colon), tissues with low self-renewal (such as the liver, brain, and bladder) appear to have a higher frequency of TERT promoter mutations^77^.

### 3.3. TELOMERASE AND SARCOMAS

Cell division in normal cells results in cell cycle arrest and death associated with senescence, which lack telomerase activity, by shortening chromosomal telomere ends. Telomerase is regularly and routinely active in bone and soft tissue sarcomas but not, in normal tissues. Telomere elongation induced by telomerase activation allows telomerase-positive sarcoma cells to proliferate indefinitely, even after cell division^109^. In the majority of cancers (85-90%), telomerase is used to preserve telomere length. The TERT gene, which encodes the catalytic portion of the telomerase riboprotein complex, has recently been found to be subject to recurrent mutations, which are the key mechanisms of telomerase activation^54^. As a malignant bone tumor, CS is the second most prevalent kind. Activating TERT mutations have recently been described as a common mutation in high-grade CS. They examined the predictive value of TERT promoter mutations in 241 CS patients gathered during a 24-year period (1994∼2017). Subsequent to the microdissection of 135 CS in 106 individuals, the TERT promoter was sequenced with information from the precursor cohort. Forty-five percent of patients had TERT promoter mutations at -124 C > T, which were substantially linked to shorter metastasis-free life, disease-specific survival, and higher tumor grade. Furthermore, tumors with TERT promoter mutations are connected to a more combative metastatic pattern. Patients having primary tumors of the wild type have shorter survival times, and metastatic mutations indicate tumor progression^110^. The genetic features of multiple sclerosis include a high frequency of hotspot mutations (C228T or C250T) in the promoter region of TERT, which encodes the TERT protein, and FUS-DDIT3 or EWSR1-DDIT3 gene fusion. The former triggers telomerase reactivation, a telomere maintenance process^14^. According to one study, TERT promoter mutations were frequently recurrent in MLS (29/39; 74%) and were not linked to the phenotype (myxoid vs. round cell variation), tumor grade, tumor location, median age, or sex of the patients in the current MLS series. This indicates that telomere maintenance, particularly in MLS, is significantly influenced by increased telomerase expression^111^. Previous studies examined the relationship between telomere length and TERT promoter mutations in the MLS sarcoma subtype. TERT promoter mutations most likely develop in a fraction of patients with MLS, with critically short telomeres as a subsequent event, most likely reflecting a compensatory mechanism that enables telomeres to lengthen or remain. Further research is required to fully examine the effects of telomere length and TERT promoter mutations on clinical outcomes, more research is required^87^. LMS do not exhibit TERT promoter mutations. However, TERT promoter mutations are, merely one mechanism of telomerase activation. It is unknown whether telomerase activity in LMS is triggered by other pathways^54^. Most people malignancies, including bone and soft tissue sarcomas, frequently exhibit active hTERT expression^112^. High-grade conventional CS frequently harbor mutations in the hTERT promoter gene. TERT promoter mutations appear to be key factors in the development of CS^113^. High tumor grade, metastatic disease, and mortality were all significantly correlated with the TERT promoter mutation, which was a poor prognostic indicator^110^, ^113^. TERT promoter mutations are simple to evaluate, and offer considerable promise as reliable clinical prognostic indicators and prospective therapeutic targets for telomerase-targeting strategies. In situations where morphology is equivocal, TERT promoter mutation analysis may be used as an adjunct to regular pathology to identify high-grade regions^110^. According to previous studies, TERT is a strong risk factor for OS and may promote cancer growth and spread. Diverse cell death pattern-related signatures for predicting prognosis and drug sensitivity in OS patients. In patients with OS, telomerase expression in the initial tumor samples is linked to lower progression-free and overall survival^114^. Sensitive and reliable telomerase activity analysis is essential for the clinical diagnosis, treatment, and prognosis of OS^115^.

## 4.TREATMENT OF SARCOMAS TARGETING TELOMERES AND TELOMERASE

Targeted therapies are becoming increasingly important in oncologists' toolkits as the molecular processes that cause sarcomas have been identified. Future clinical trial designs must consider this molecular understanding and make use of carefully chosen biomarkers to help determine the best targets for inhibition, as well as predictive and prognostic aspects^116^. STS is an uncommon and diverse group of tumors. Therefore, our therapeutic strategy is neither histology-specific nor generally standardized. The variability of these tumors has hampered the development of evidence-based treatment methods^49^. Modern approaches such as immunotherapies and medications that target epigenetic pathways are now being researched to enhance the results for this group of people^117^. Currently, children and adolescents are most frequently affected by OS, which is a very aggressive bone tumor^118^. Neoadjuvant MAP chemotherapy, together with further rounds of chemotherapy administered after the patient has recovered from surgery (adjuvant postsurgical chemotherapy), is the most popular treatment for young patients with resectable OS^61^. However, standard chemotherapy regimens are frequently ineffective in treating OS, and the use of drugs for tumor chemotherapy can have several negative side effects^118^. Over the past four decades, patient outcomes have remained consistent when localized OS is treated with established three-drug chemotherapy regimens^119^. Approximately 40% of OS cell lines test positive for ALT, making them resistant to the majority of chemotherapy treatments, and the lack of anti-cancer treatments that target ALT makes treating OS in the clinic even more difficult^118^. Targeting telomerase has several benefits over most other cancer targets, including potential cancer stem cells, due to its ubiquity, importance, and selectivity for cancer cells. Although there is a telomerase-independent ALT mechanism for maintaining telomeres in cancers and cell lines where telomerase is inactive or suppressed, some studies have indicated that ALT cells are less biologically active than telomerase-positive cancer cells and may be more vulnerable to medications that cause oxidative stress^120^. Telomerase is a prime candidate for cancer therapy^121^. One effective anti-cancer drug for the management of bone and STS is an oncolytic adenovirus that targets telomerase^122^. Telomerase can be targeted through immunotherapies that target TERT tumor-associated antigens, small-molecule inhibitors, or oligonucleotides that directly bind telomerase and prevent telomere extension 103.

### 4.1. TARGETING TRF1

Cancer cells are eliminated by TRF1 inhibitors like ETP-47037 and ETP-47228 because they obstruct TRF1 binding at the telomere point and stop the formation of the shelterin complex^26^. Recently, a new TRF1-targeting method was developed for inducing telomere dysfunction. This method is independent of the telomerase activity and length. Although further clinical studies are required to determine the effectiveness of pharmacological inhibitors that uncap telomeres in human cancer, these approaches provide new avenues for the development of anti-cancer drugs^123^. An innovative method for addressing telomeres in cancer cells was made possible by a study that produced a proof-of-concept, TeloTAC, which selectively breaks down TRF1 and TRF2 and interferes with telomerase function. Compared to normal cells, the cytotoxicity of the prototype TeloTAC 15b was much greater because of the effective degradation of TRF1 and TRF2. Therefore, the TeloTAC technique is useful as a new PROTAC approach that targets cancer. However, TeloTAC has several limitations due to the nature of these nucleotide oligomer-based designs, particularly in terms of potential clinical translation^124^.

### 4.2. SMALL MOLECULES AS TELOMERASE INHIBITORS

Currently, few effective hTERT inhibitors are available on the market. Among the active site inhibitors unique to hTERT with the greatest potential yet is BIBR1532, also known as 2-[E]-3-naphtalen-2-yl-but-2-enylylamino]-benzoic acid. A number of BIBR1532 research have revealed that telomerase is dose-dependently inhibited by increasing doses of BIBR1532, whereas no discernible effects were observed in normal human cells^125^. The artificial mixed-type noncompetitive non-nucleoside telomerase inhibitor BIBR1532 targets the telomerase catalytic subunit hTERT, with an IC50 value of 93 nM. Although its precise mode of action remains unknown, it may prevent telomerase translocation. Cancer cells undergo apoptosis and senescence in response to BIBR1532^126^. BIBR1532 selectively hinders DNA substrate elongation during template copying rather than the fundamental steps of template copying. Long-term therapy with BIBR1532 caused considerable telomere shortening and an 80% reduction in TA in CS cell lines 125. The results showed that treatment with BIBR1532 led to telomerase inhibition, reduced telomere length in telomerase-positive CS cells, and decreased their growth capability^127^. A 13-mer oligonucleotide called GRN163L(imetelstat) functions as a direct antagonist of the telomerase RNA template, attaching to the active site of hTER with high specificity and affinity to completely suppress the enzyme^125^. It has also been demonstrated both in vivo and in vitro that GRN163 is a potential anti-cancer agent that targets telomerase activity. GRN163 causes tumor cells to shorten their telomeres, which is followed by cellular senescence or apoptosis^128^. Imetelstat has shown the most promise when combined with other chemotherapeutic drugs, such as trastuzumab, 3-aminobenzamide (3AB) or traditional radiation therapy 129. GRN163L has been used in several clinical trials 125. Subsequent research on imetelstat is needed to explore its potential for the synergistic targeting of hTERT when used in conjunction with other medications, such as small-molecule inhibitors, which simultaneously minimize adverse effects and target other sources of cancer aggressiveness 129. The ideal dose of imetelstat in phase II for kids with solid tumors that come back or are resistant was investigated in a previous study^130^. Imetelstat increases telomerase suppression by the HSP90 inhibitor alvespimycin. The capacity of the HSP90 inhibitor alvespimycin to enhance the telomerase inhibitor's impact was evaluated as the chaperone molecule HSP90 promotes the assembly of telomerase proteins. However, the combination treatment group showed the strongest effect 131.* K Trieb et al*. reported that hyperthermia may offer a fresh adjuvant tactic for treating patients with OS by inhibiting telomerase activity. These findings demonstrate that OS cells undergo telomerase activity that is reversibly downregulated in response to heat^132^.

### 4.3. G-QUARDRUPLEX STABILIZERS AS TELOMERASE INHIBITORS

In order to prevent invasion and annealing of telomeric single-stranded DNA, the development of a G-quadruplex in the 3′-terminus of telomeric DNA may be useful as an anti-ALT cancer treatment. Therefore, a chemotherapeutic agent that precisely targets the telomeric G-quadruplex, in addition to having strong anti-tumor effects, may be more beneficial in the treatment of OS^118^. G-4 stabilizers offer a great deal of promise as anti-cancer therapies because of their high tolerance in in vivo experiments and ability to disrupt telomere structure by blocking the telomerase-binding proteins TRF2 and POT1^126^. One of the best compounds for telomeric G4 stabilization is the pentacyclic acridine compound RHPS4^133^. In addition to other modifications, aggressive tumors overexpress K^+^ membrane ion channels that lower the roughly 60 mM intracellular K^+^ concentration. Studies conducted in vitro have demonstrated that at such K+ concentrations, G4 structural alterations are still imperceptible, but their overall stability is decreased. Therefore, accurate and effective G4 DNA stabilization and anti-cancer medication administration could possibly be achieved using novel anthraquinone chemicals linked to distamycin analogs^134^. Quinazoline molecules specifically stabilize G4 complexes in vitro and in human cells. But no successful clinical trials have yet been the result of it^135^. Chemical substances known as G4 ligands maintain G4s stable. *Streptomyces anulatus* produces the natural G4 ligand, telomestatin, which binds to G4s and prevents telomerase activity. Telomestatin induces telomere dysfunction in cancer cells by removing TRF2 and POT1 from the telomeres^136^. In photodynamic treatment (PDT), TMPyP4 is a unique synthetic water-soluble photosensitizer with significant electron-hole transfer capacity. An inflammatory microenvironment linked with tumors is more prevalent in OS. The results showed that TMPyP4 could induce human telomeres and FAK G-quadruplexes both in vitro and in vivo. These findings indicate that TMPyP4 is a better option than cisplatin for treating OS^118^.

### 4.4. AGENTS AS TERT INHIBITORS

Human TERT contains 1,132 amino acids. The early observation that bulk tumors are often telomerase positive serves as the foundation for TERT immunotherapy. A significant finding from more recent research is that TERT plays crucial roles in every step of carcinogenesis, and its expression occurs at all stages of the cancer process, from early CSCs and/or tumor-initiating cells to advanced metastatic cancer cells^96^. TERT-based immunization has been demonstrated to results in tumor regression in several preclinical models. TERT-derived peptides may be identified by cytotoxic CD8+ cells in a limited MHC class I way^137^. Immunotherapies targeting the hTERT antigen have shown promising results and may soon be used to treat cancer^126^. Studies have investigated the effects of short hairpin RNAs (shRNAs) targeting hTERT on OS cell death and proliferation. It has been shown that by decreasing telomerase activity, shRNA targeting the hTERT gene may suppress cell growth and encourage OS cell death. Further studies are required to determine whether hTERT-shRNA can be used in conjunction with radiotherapy and other therapies to treat OS^138^. The flexible genome editing method CRISPR/Cas9 has enormous potential for cancer treatment^139^. Wen et al. found that inactivation of TERT is a promising cancer treatment. The scientists discovered that altering TERT affected tumor cell viability both in vitro and in vivo using the CRISPR/Cas9 gene-editing technique. Crucially, the CRISPR/Cas technology is capable of eliminating cancer cells, making it a cutting-edge and promising anti-cancer treatment that doesn't have the negative consequences of other established techniques^140^.

### 4.5. TELOMERASE-SPECIFIC ONCOLYTIC VIROTHERAPY

A potential therapy for solid tumors is oncolytic virotherapy, which employs viruses engineered to only multiply in cancer cells^141^. OBP-301-based, OBP-401-based, and OBP-702-based oncolytic virotherapies for the treatment of sarcomas with an invasive character would be a potential anti-cancer approach. Upon infection, OBP-301 penetrates and multiplies in tumor cells with telomerase activity because of the activation of the hTERT promoter. Subsequent stimulation of autophagy and cell lysis caused by excessive viral replication causes the virus to disseminate to nearby tumor cells. OS cells are more sensitive to doxorubicin and cisplatin in the presence of OBP-301. The upregulation of E2F1 and microRNA-29 by the adenoviral E1A protein results in the inhibition of the anti-apoptotic myeloid cell leukemia 1 protein, which is the biological mechanism responsible for OBP-301's improvement of chemosensitivity. Therefore, a potential anti-cancer approach for the treatment of OS involves a combination of OBP-301 plus chemotherapy^109^. OBP-301 was shown to be safe when used as monotherapy for patients with advanced solid tumors, such as LMS and neck sarcoma, according to Phase I clinical research conducted in the US^142^. They demonstrated the diagnostic potential of the oncolytic adenovirus OBP-401 expressing green fluorescent protein (GFP) for evaluating the sensitivity of bone and soft tissue sarcomas to viral therapy^112^. The effectiveness of fluorescence-guided surgery using OBP-401 for various kinds of solid tumors, such as OS and STS, has been demonstrated in vivo using preclinical tests. Specifically, in orthotopic xenograft tumor models, including human OS and fibrosarcoma cells, OBP-401-based FGS considerably decreased lung metastasis and local recurrence compared to bright-light surgery^109^. The GFP expression that OBP-401 mediated showed a strong correlation with OBP-401's therapeutic benefits for soft tissue and bone sarcomas in humans. ATRX is among the genes that solid tumors most frequently have mutations in, and STS are particularly prone to this mutation^143^. Sarcomas with ATRX deficiency and aberrant CGAS/STING signaling are sensitive to oncolytic herpes virus therapy^143^. An oncolytic adenovirus called OBP-702 expresses p53 and significantly activates the p53 signaling pathway. Compared with OBP-702 and OBP-301, they cause a deeper anti-tumor effect in OS cells by promoting autophagy and apoptosis. In OBP-702-infected cells, p21 suppression, induced by E1A-mediated miR-93/106b overexpression resulted in p53-mediated cell death and autophagy. A damage-regulated autophagy modulator (DRAM) was activated in response to p53 overexpression, which improves adenovirus-mediated autophagy. Furthermore, in an orthotopic MNNG/HOS xenograft tumor model resistant to OBP-301, OBP-702 inhibited tumor development. These findings imply that OBP-702-mediated p53 transactivation is a viable anti-cancer strategy that triggers autophagic and apoptotic cell death pathways in human OS cells through the control of DRAM and miRNA^144^. OBP-502 is a telomerase-specific replication-competent oncolytic adenovirus that binds to integrins and causes cell death. Telomerase-specific oncolytic virotherapy is a viable anti-cancer strategy that enhances the effectiveness of the PD-1 blockade in OS^145^. Oncolytic virotherapy targeting telomerase is currently used in clinical settings to treat patients with cancer. Oncolytic adenoviruses driven by the hTERT promoter are anticipated to provide innovative treatment options^109^, ^122^.

## 5. CONCLUSION AND PERSPECTIVE

The link between telomeres, telomerase, and many cellular functional pathway abnormalities associated with cancer is complex^146^. Telomere changes and telomerase reactivation occur frequently in sarcomas. In the future, telomeres and telomerases may be used as prognostic markers for sarcoma, and these markers may be used in the staging of patients with sarcoma for improved treatment. New treatment methods and ideas will provide a theoretical basis and direction for clinical research, which will be beneficial to overcome the challenges and shortcomings in the present treatment options. Furthermore, they will improve accuracy and effectiveness of prognosis and treatment. Despite the promise of this approach, its clinical application faces significant hurdles. Firstly, a limited number of clinical studies have been conducted thus far. Secondly, potential safety concerns and other unforeseen issues remain to be comprehensively investigated. In essence, substantial work is required before widespread clinical use.

## Figures and Tables

**Figure 1:**
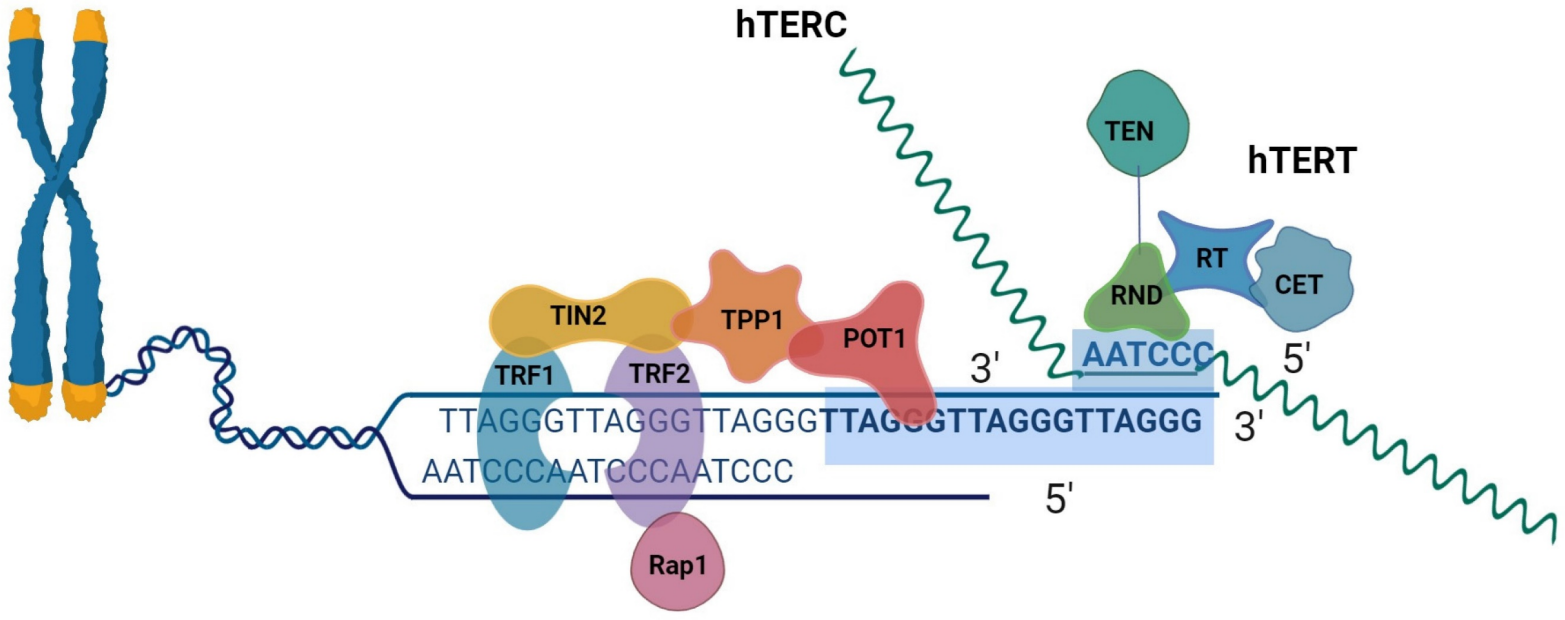
Schematic representation of telomeres, the shelter complex, and telomerase.

**Table 1:** Sarcoma-associated activation of telomerase

Tumor type	Phenotype	Incidence rate (%)	Mechanism	Reference
OS	hTERT positive	32		114
	ALT	11-66	TOP3A amplification	114, 147-151
CS	hTERT mutation	43-45	The TERT promoter mutation at -124 C>T	35, 110, 152
STS	telomerase activity	7-81		148, 153-156
EWS	hTERT positive	12-84	fusion proteins EWS-ETS	151, 152, 157-159
LPS	hTERT positive	18-64		99, 160
	ALT	25.9		149
DDLPS	ALT	80	loss of either ATRX or DAXX	65, 160
MFH	hTERT positive	22-90	p38 MAPK	151, 157
MLS	hTERT mutation	74-77	fusion proteins FUS-DDIT3 or EWSR1-DDIT3	14, 111

Abbreviation: Malignant Fibrous, MFH

**Table 2:** Targeting telomeres and telomerase in sarcomas

Target	Drug	Mechanism	Reference
TRF1	ETP-47037 and ETP-47228	Impede TRF1 binding at the telomere point and stop the shelterin complex formation	26, 123
TRF1 and TRF2	TeloTAC	Degradation of TRF1/2 to induce collapse of the shelter-in complex and subsequent disruption of telomerase binding to telomere ends	124
Telomerase	BIBR1532	Non-competitive binding to the hTERT active site to inhibit telomerase	125-127
	GRN163 (Imetelstat)	Acts as a direct telomerase RNA template antagonist	125, 130, 131
	RHPS4 and TMPyP4	Disruption of the shelter-in complex, preventing telomerase from binding to telomeres	118, 133-136
	OBP-301, OBP-401, OBP-502 and OBP-702	Induction of telomerase-positive tumor cell death under the control of the hTERT promoter	109, 112, 141, 142
TERT	GV1001, GRNVAC1	Vaccine against TERT	124
